# The role of phone-based triage nurses in supporting older adults with multimorbidity to digitally self-manage – Findings from the ProACT proof-of-concept study

**DOI:** 10.1177/20552076221131140

**Published:** 2022-10-09

**Authors:** Julie Doyle, Patricia McAleer, Cora van Leeuwen, Suzanne Smith, Emma Murphy, Myriam Sillevis Smitt, Mary Galvin, An Jacobs, Lorraine Tompkins, James Sheerin, John Dinsmore

**Affiliations:** 1 NetwellCASALA, 8817 Dundalk Institute of Technology, Dundalk, Ireland; 2 Imec-VUB-SMIT, Brussels, Belgium; 3 Trinity Centre for Practice and Healthcare Innovation, 8809 Trinity College Dublin, Dublin, Ireland; 4 School of Computer Science, 8819 Technological University Dublin, Dublin, Ireland; 5 Department of Design Innovation, 8798 Maynooth University, Maynooth, Ireland; 6 Home Instead Senior Care, Dublin, Ireland

**Keywords:** Multimorbidity, digital health, self-management, older adults, clinical triage, integrated care

## Abstract

**Background:**

Achieving patient-centred care necessitates supporting individuals to have more involvement in the self-management of their care. Digital health technologies are widely recognised as a solution to empower more effective self-management. However, given the complexity of multiple chronic condition (multimorbidity) management, coupled with changes that occur as part of the normal ageing process, human support alongside digital self-management is often necessary for older people with multimorbidity (PwM) to sustain successful self-management.

**Methods:**

The aim of the study was to explore the role played by a clinical, nurse-led telephone triage service in responding to alerts generated by older adults using a digital health platform, ProACT, to self-manage multiple chronic conditions over a period of 1 year. Semi-structured interviews with participants with multimorbidity were carried out across four time points during the trial, while interviews and focus groups were conducted with triage nurses at the end of the trial. Thematic analysis was conducted on the resulting transcripts.

**Results:**

Themes found in the data include the work of triage nurses; the benefits of triage support; tensions such as anxiety due to patient monitoring; and the relationship between triage nurses and participants.

**Discussion:**

This work contributes to an understanding of how older adults with multimorbidity and triage nurses collaborate in multiple chronic disease self-management. Findings are discussed within the context of Hudon et al.'s patient-centred care framework and indicate that patient-centred care was achieved, with both PwM and triage participants reporting positive experiences, relationships and several benefits of the triage support alongside digital self-management.

## Introduction

### Background

There is a vast body of research on the importance of patient-centred care and a number of frameworks and models exist to help guide and shape how patients and healthcare professionals (HCPs) can collaborate to achieve optimal outcomes, particularly in the context of chronic disease care.^1^ This is increasingly important given that global populations are ageing, resulting in higher rates of chronic disease, including multimorbidity and the presence of two or more chronic conditions.^2^ There are an estimated 50 million people in the EU living with multimorbidity^3^ and it is becoming increasingly prevalent in ageing populations.^4^ Hudon et al.^1^ proposed a framework of patient-centred care that combines the model proposed by Stewart et al.,^5^ with a model of the doctor-patient relationship developed by Mead and Bower.^6^ Hudon et al.'s framework identified four dimensions relevant to patient-centred care namely; the patient as a person (how the person experiences their illness), the biopsychosocial perspective (considering the whole person), the therapeutic alliance (patient-doctor relationship) and sharing power and responsibility (common ground). A patient-centred approach to care calls for a move from the traditional paternalistic model of care to one that favours the patient as having a more prominent role in decisions about their care. According to Pichon et al.,^7^ *“patient experience and expertise are not consistently acknowledged in the current medical model or the traditional role of the patient, but are in fact key to patient empowerment and enable pragmatic handling of uncertainty in the intricate day-to-day contingencies of self-management”*. Involving individuals as equal collaborators in their care necessitates empowering them to self-manage their health and wellbeing.

Self-management, defined as the actions taken by an individual to manage symptoms, treatment, emotions and lifestyle changes as part of living with a chronic condition,^8^ is an important task to ensure good health.^9^ However, there are many challenges people with multimorbidity (PwMs) face in self-managing. People can experience symptoms (e.g., fatigue and breathlessness), age-related declines (e.g., reduced mobility), pain and depression, which can all severely impact or prohibit engagement with self-management.^10^ Those with multimorbidity also tend to report insufficient knowledge and limited support to effectively self-manage, as well as a lack of integration of care amongst their various healthcare providers.^10–12^ It is not surprising, therefore, that adherence to treatment and self-management for those with chronic conditions, and particularly multiple chronic conditions, tends to be low,^4,13^ with additional factors that impact adherence including age, information on treatment plans, knowledge of medication regimens and self-perception of quality of life.^14^

Digital health technologies have the potential to support effective self-management and various digital approaches and strategies have been explored in the literature demonstrating the value of such technology.^15–17^ While technology has the potential to support many self-management tasks and should strive to support the autonomy of the person self-managing, human support is also important, particularly in achieving person-centred care and promoting adherence to self-management and digital interventions.^10,18,19^ Indeed, individual responsibility for self-management without support from an HCP can be detrimental.^7^ Expert or clinical support can be important where guidance or reassurance is needed or to provide emotional support for the person.^19^ Furthermore, individuals often prefer to partner with their HCPs, rather than self-manage autonomously.^7,20^

However, research also points to the many challenges in the relationships between patients and HCPs, particularly within the context of multimorbidity, which can negatively impact self-management efforts.^21–23^ Challenges reported by patients include limited access to, and poor communication with, their individual HCPs; having no one to answer questions or concerns; not being listened to during consultations; a lack of knowledge on how to self-manage due to confusing and contradictory information from multiple HCPs; and differences in priorities and values between patients and HCPs.^10,12,23^ PwMs also complain about poor or no communication between their different HCPs, which results in a reliance on the person to be responsible for all communication with providers, and difficulties in prioritising different parts of their treatment plans.^21^ Patients have also reported a lack of trust in their HCPs, which can result in a reluctance to share information with them.^22,24^ PwMs would appreciate more time during clinical visits to discuss their values so that these could be considered in an HCP's care plan and desire more personal and intimate interactions with HCPs as well as more guidance on how to articulate their values.^23^ PwMs would also like to be able to ask questions and find answers to their health concerns, want to be listened to, and want doctors to appreciate what needs they, as PwMs, consider important to be met.^10^ Further to these PwM reported preferences, research involving older adults with multiple cardiac conditions found participants experienced benefits from interactions with their HCPs, describing enjoyable, sometimes personal relationships and expressing gratitude for the level of care received. These same participants did not engage in the use of technology to manage their care, perhaps preferring human support.^25^

HCPs also experience numerous challenges in caring for PwMs, often reporting limited time to interact with patients, create formal integrated care plans or conduct medication reviews, as well as feeling burdened by having to monitor patient data.^7,12,26–28^ There is frequently dissonance between what patients’ desire and what HCPs typically can, or are willing to, provide in terms of support for managing conditions. Given the importance of support as an enabler of self-management, solutions are needed to address this gap between patient expectations and what HCPs can provide. The presence of a care coordinator who monitors readings and provides alerts when anomalies are recorded has been shown to enhance engagement and confidence in self-management activities with digital interventions.^26,29,30^ Conversely, there is also evidence to suggest where there is the awareness that symptoms are being monitored, there may be a *reduction* in self-management behaviours. However, dependence on a care coordinator, to notice anomalies and provide an alert, has the potential in absolving the person of responsibility to take action when readings are outside the acceptable range unless alerted by the care coordinator.^26^ This concern is especially highlighted by HCPs and is one reason given for their reluctance to take on routine responsibility for such monitoring oversight for their patients.^26^

In this article, we present findings from a longitudinal study whereby 120 older PwMs were recruited, across two European countries, to use a custom-built digital health platform ProACT^31^ to self-manage their conditions with clinical oversight by a team of triage nurses in each country. The triage service aimed to respond, via a phone call, to alerts in symptom readings, thus providing a safety net for participants self-managing. However, as the trial progressed, it was clear that the triage teams played a much more important role than had been anticipated. This article outlines qualitative findings from both PwM participants and triage nurses in relation to the role played by the triage service in supporting digital self-management. Themes include the work of triage nurses; the benefits of triage support; tensions such as anxiety due to patient monitoring; and the relationship between triage nurses and participants. The contributions of this work include an understanding of how PwMs and telephone triage nurses collaborate in multiple chronic disease digital self-management. As healthcare providers and healthcare systems are a long way from providing true patient-centred, integrated and collaborative care, particularly for those with multimorbidity, we argue that digitally-supported phone-based clinical triage support can fill this gap.

## Methods

This work took place within a larger action research proof-of-concept trial to explore the use of technology to support self-management and enhanced integrated care for older PwMs. The full study protocol has been published elsewhere.^32^ The study received ethical approval from three ethics committees in Ireland and four in Belgium.

### The ProACT digital health platform

**Figure 1.**
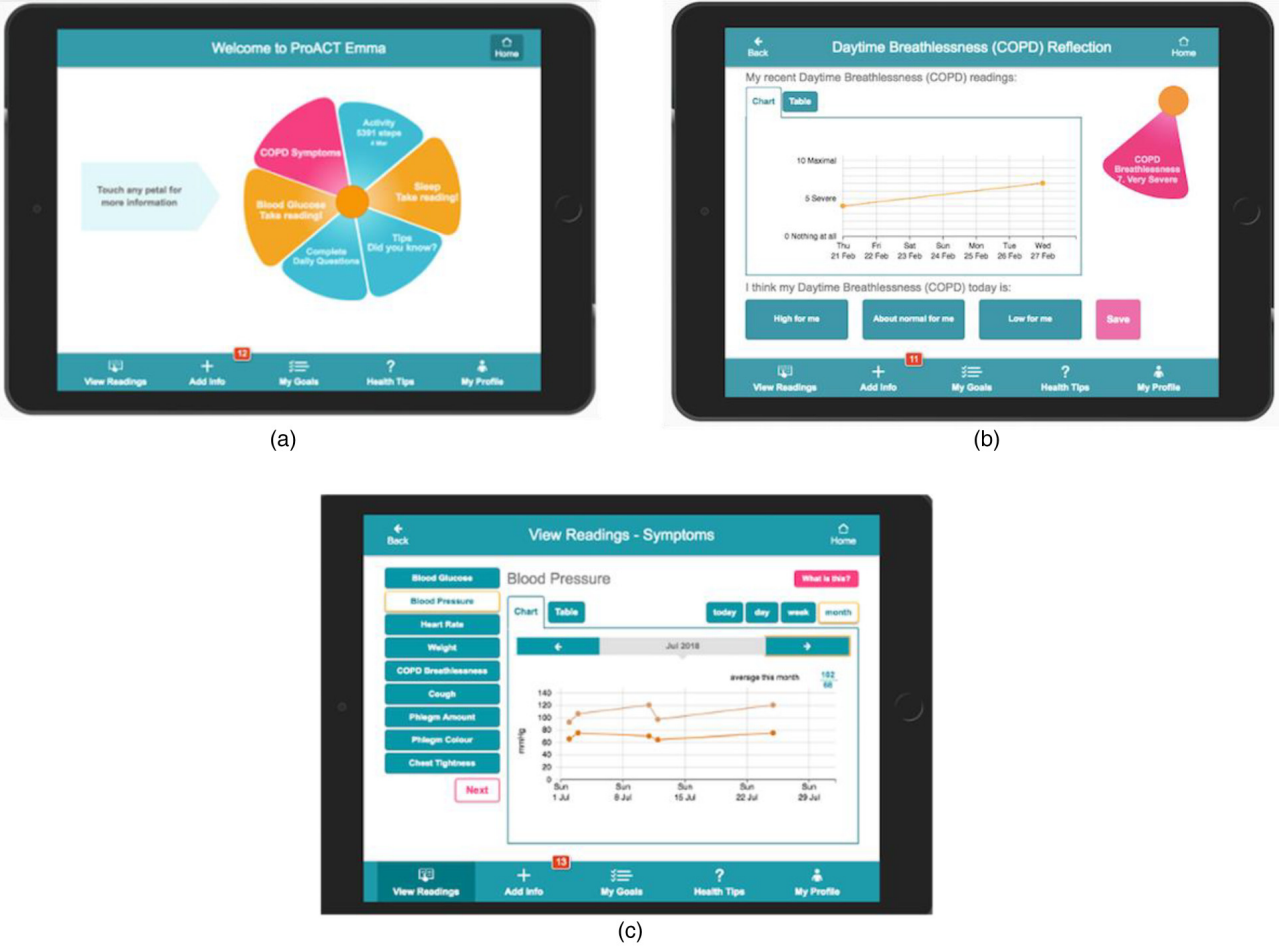
Participant CareApp showing (a) dashboard highlighting current status, (b) reflecting on data and (c) viewing readings.

The ProACT platform supports PwMs in the self-management of their multiple chronic conditions. The full ProACT platform, including all its backend components, has been described elsewhere,^31^ along with the process involved in co-designing the platform with end users. From the PwM's point of view, ProACT consists of sensing devices for measuring symptoms (e.g., blood pressure, blood glucose and blood oxygen level) and wellbeing (e.g., weight, activity and sleep) parameters. The ProACT CareApp (Figure 1) is used to view data over time, self-report on health and wellbeing (e.g., breathlessness and mood), view education related to conditions and how to use the CareApp and devices, set goals in relation to physical activity and add people to their care network and share data with them.

**Figure 2.**
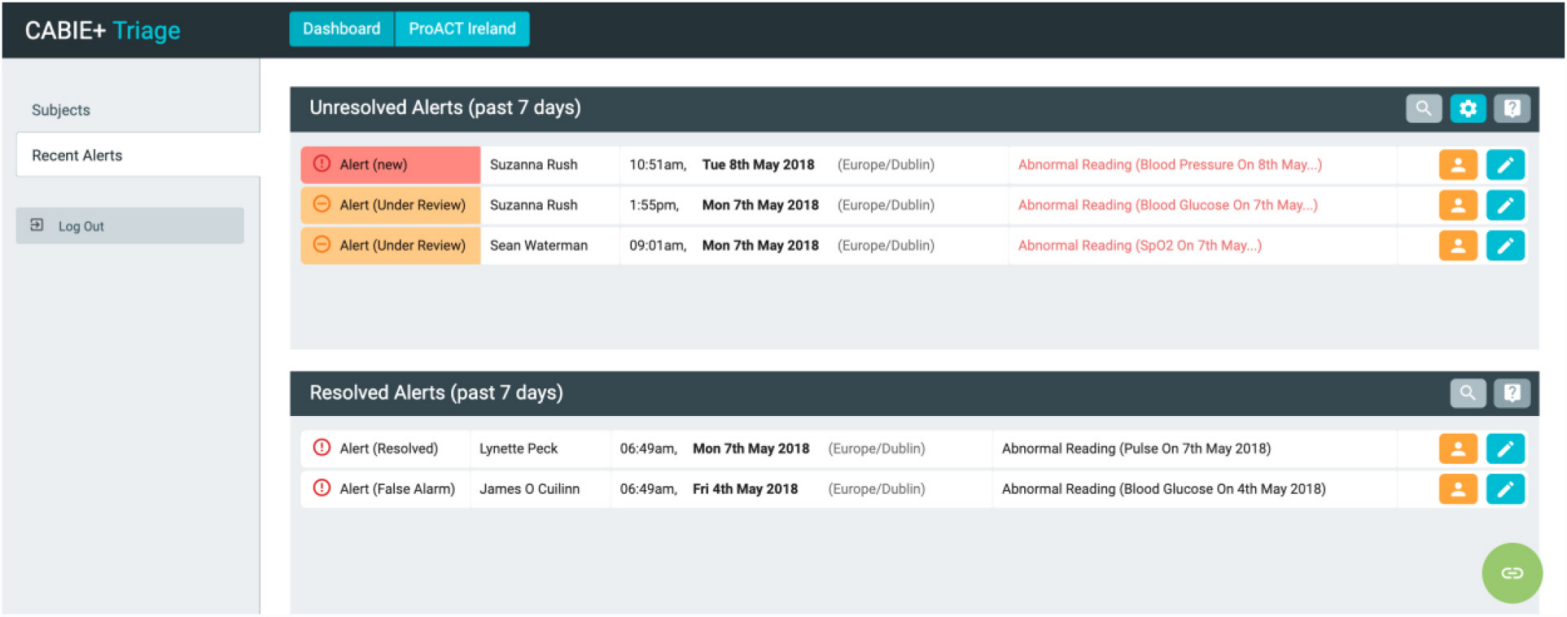
Triage interface dashboard with alerts (names shown are not real).

**Figure 3.**
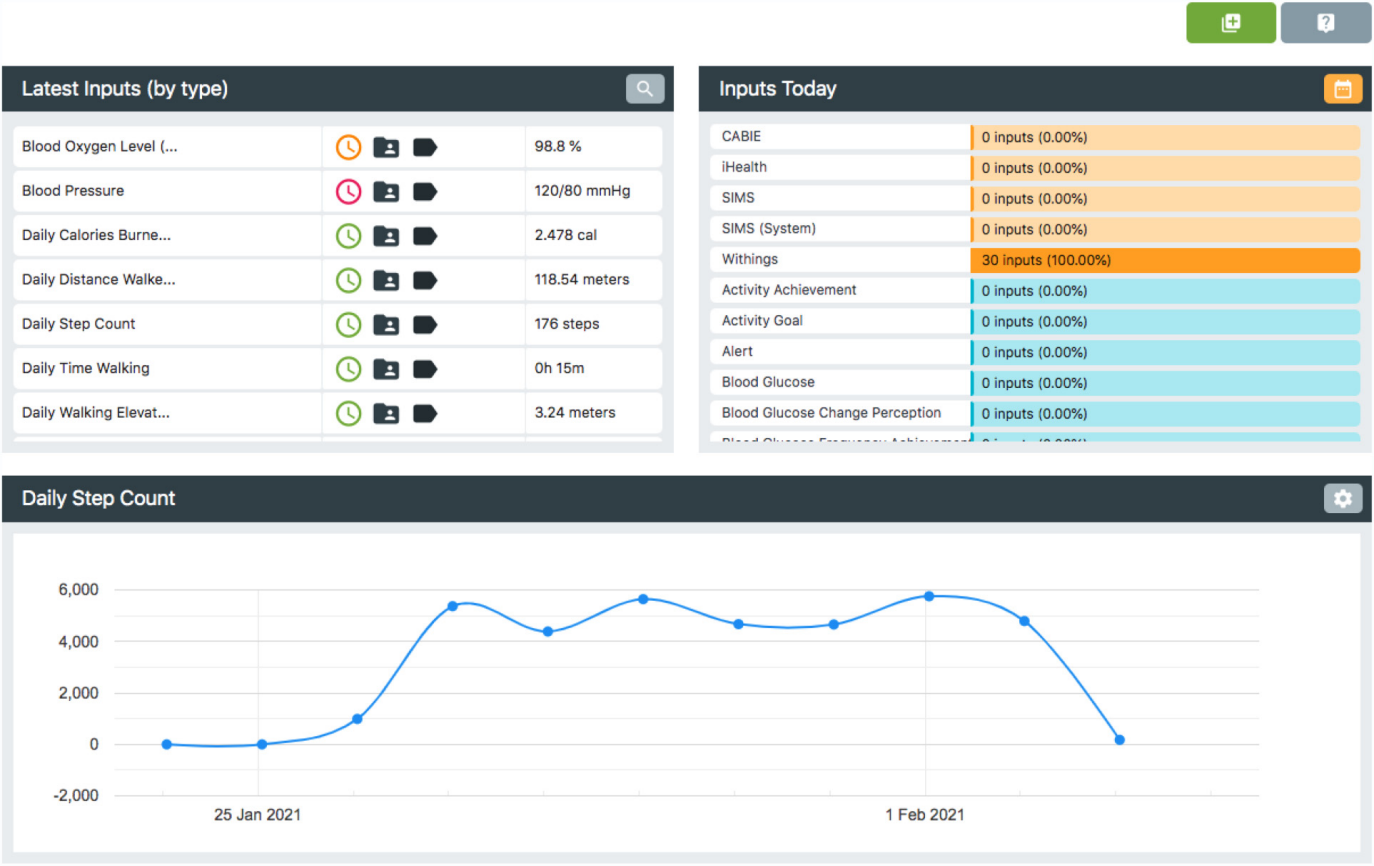
Inspect feature allows the querying of data for each participant and shows their most recent inputs.

Triage nurses use a custom-designed application (SIMS-triage) to view and respond to alerts from data collected by PwMs in the home. Alerts are generated when thresholds are breached for different parameters, defined within the system. For example, an alert for high blood glucose is a reading over 14 mmol/L (configurable per participant). Participant placement on the triage alert list is prioritised by alert status – i.e., those with a more urgent ‘red alert’ status appear first (Figure 2). A tag also appears alongside the alert, indicating if it is a new alert or currently under review by a nurse. Within the dashboard, the nurse can also view recently resolved alerts and the PwM's health and wellbeing data (Figure 3). This provides the triage team with a holistic picture of the PwM's status prior to calling them to discuss an alert. Nurses can also create notes in relation to alerts, allowing for a rich description of the context linked to the alert readings.

### Inclusion criteria and participant recruitment

In total, 120 PwMs consented to take part, 60 in both Ireland and Belgium, respectively. PwM participants were recruited through several channels, including social groups for older adults, condition support groups, social media, radio and local newspaper advertising, a formal care organisation, HCPs and living lab organisations. Inclusion criteria were that participants were age 65 or over and had two conditions from the following: chronic obstructive pulmonary disease (COPD), congestive heart failure (CHF), chronic heart disease (CHD) and diabetes. It should be noted that one participant in Belgium was 60 years old. This participant had initially recorded an incorrect date of birth on the screening documentation, and this was not discovered until after the participant had already begun the trial. This person was excluded from the core analysis presented in this article. The triage organisations in each country were selected following a tendering process. In Ireland, a team of four triage nurses provided the service to PwMs for the duration of the trial, while there was a team of six nurses in Belgium, with two nurses fulfilling the primary role. The remaining four nurses had access to and training on the ProACT platform and filled in as a triage support if the two primary nurses were unavailable.

### Procedures

Each PwM was provided with an iPad, a smartwatch to monitor sleep and activity, a digital weight scale and a digital blood pressure cuff. Those with diabetes were provided with a blood glucose monitor (glucometer) whilst those with COPD were provided with a pulse oximeter to measure SpO2 levels. In the initial visit to the PwM's home, the devices were set up by the researcher and training was provided on how to use the devices. A second visit, approximately 1 week later, introduced the ProACT CareApp to PwMs and further training was provided. Each PwM received a detailed paper-based training manual, while training videos on how to use the devices and the CareApp were also available in the CareApp. During the trial, PwM data was monitored by the clinical triage teams in each country. Triage staff determined a protocol for alerts and escalation procedures in case of an alert.

Alerts were set within the SIMS triage platform described above. These were initially set at a global level (e.g., the same blood glucose thresholds were set for all diabetic participants) and as the trial progressed, individualised threshold levels for alerts were established based on the PwM's usual range or input from their HCP. A list of each participant's medications and their additional comorbidities were collected by researchers and were input into the SIMS triage platform, where they were available for triage nurses to view. These were updated as necessary throughout the trial by triage staff, based on their phone conversations with participants. Triage staff responded to alerts between 9 a.m. and 5 p.m., Monday to Friday (hours were limited due to the project budget). In addition to monitoring alerts, the triage team also conducted monthly check-in phone calls with participants. While triage nurses advised participants on what action they should take (e.g., visit their GP or contact their consultant), based on their data and phone conversations, it was the participants’ responsibility to act on this advice. Triage nurses had no direct contact with the PwM's HCPs, as the focus of this study was to explore supported self-management, with the PwM ultimately having responsibility for making decisions in relation to following advice. However, as is outlined in the ‘Findings’ section, there were instances where triage nurses called the ambulance service to take PwMs to the accident and emergency, where their condition was deemed serious enough to warrant this. Participants were made aware that they were responsible for following up with their HCPs, as well as the hours of triage, both through their project information sheet and through a weekly pop-up message in the CareApp. In addition, participants were advised that the triage service was not a replacement for usual care and that they should seek medical advice, as they usually would, for any health concerns. Participants acknowledged understanding this when signing the trial consent form, which was also explained verbally. A helpdesk number was provided, that could be called in the event of technical issues arising or if participants wanted to request further training. A researcher was available to answer calls between 9 a.m. and 5 p.m. Monday to Friday. Outside of these hours, participants could leave a voicemail message for follow-up by the research team or send a ‘call me’ message for the research team through the CareApp.

### Data collection and analysis

A semi-structured focus group (Ireland) and interviews (Belgium) were conducted with the triage nurses at the end of the trial, at their place of work (conducted by authors JDoy, CvL and MSS). Four nurses took part in the focus group in Ireland while three nurses were interviewed in Belgium. The same protocol and questions were asked in both Ireland and Belgium. Questions were structured under four broad topics including (1) General questions (describe a typical working day (before using ProACT), a typical working day with ProACT and ProACT clients, previous experience with digital health systems); (2) Monitoring symptoms/data (what data was assessed for an alert and for a check-up call, escalation procedures and any concerns related to trust or reliability of data); (3) Dealing with PwM clients (their perception of how PwMs were managing with ProACT, their response to the triage service; any challenges, such as participants not the following advice; any alert/exacerbation events of note that led to a clinical outcome such as a medication change); (4) Experience of using the technology (collaborative use of SIMS-triage, usability issues, requests for additional features).

Semi-structured interviews were conducted with PwMs in Ireland and Belgium at four time-points across the trial (T1-T4). All data collection with PwMs took place at the participants’ homes (conducted by authors JDoy, PMcA, CvL, SS, EM, MSS, MG, LT, AJ and JS). Interviews with PwMs explored various aspects of their experience with and usage of the platform. This article focuses on findings in relation to PwM perceptions of the triage service. All interviews and focus groups were audio recorded and transcribed verbatim.

Researchers conducted a thematic analysis (TA) on the resulting transcripts using NVivo 11 for Mac (in Ireland) and MaxQDA (in Belgium). The linear six-stage method of TA, outlined by Braun and Clarke^33^ was used by researchers in each country. An inductive approach was taken to coding, including line-by-line coding followed by a second stage of broad coding to ensure meaning and context were accounted for (conducted by JDoy, PMcA, CvL, SS, MSS, LT and JS). A sample of transcripts was double-coded in each country to confirm inter-coder reliability. The same procedures were followed in both countries, with researchers within each country and across both countries meeting regularly during the data analysis process to discuss, compare and validate codes and themes, until there was a consensus. In keeping with Lincoln and Guba's^34^ criteria for trustworthiness in the TA of qualitative data, researchers conducting the analysis had participated in a review of the literature and the trial protocol design, in addition to having prolonged regular engagement with participants over the course of the 12-month trial. This enabled persistent observation and triangulation of data for credibility. A logical and documented protocol for data collection and analysis was followed to ensure dependability and confirmability.

## Findings

Demographic data for PwM participants can be found in Table 1. In Ireland, the average age of the participants was 74.23 years (SD 6.4), and 60% (*n* = 36/60) were male. In Belgium, the average age of the participants was 73.88 years (SD 6.23), and the participants were predominantly male (*n* = 42/59, 72%). An overview of themes is presented in Table 2. There were four main themes, three of which had a number of sub-themes. As T1 represented the start of the study when participants had not yet experienced the triage service, the findings presented are from T2 to T4. For PwM participants, quotes are structured as (participant id, gender, age, inclusion conditions, timepoint and country), while triage nurse (TN) quotes are denoted by (TN, country).

**Table 1.** Demographics of PwMs in Ireland and Belgium.

**Age** mean, SD (range)	Ireland (IE)	Belgium (BE)*
	74.23 ± 6.4 (65–92 years)	73.88 ± 6.23 (65–91)
**Gender** *N* (%)		
Male	36 (60)	42 (72)
Female	24 (40)	17 (28)
**Ireland highest educational level** *N* (%)		
Some primary	3 (5.0)	
Primary	14 (23.3)	
Secondary	20 (34)	
Junior certificate	10 (16.7)	
Leaving certificate	10 (16.7)	
Diploma/cert	12 (20)	
Primary degree	1 (1)	
Postgrad	10 (17)	
**Belgium – highest education level** *N* (%)		
None/primary		1 (2)
Lower high school		14 (25)
Higher high school		12 (21)
Higher education		14 (25)
University		16 (28)
**Living status** *N* (%)		
Lives alone	25 (42)	18 (31.6)
Lives with others	35 (58)	39 (68.4)
**Marital status** *N* (%)		
Married	28 (46)	36 (64)
Living with partner	4 (7)	2 (3)
Widowed	18 (30)	7 (12)
Single (never married)	4 (7)	4 (7)
Separated	4 (7)	1 (2)
Divorced	2 (3)	5 (9)
**Employment status** *N* (%)		
Retired	55 (92)	52 (91)
Permanently sick/disabled		1 (2)
Self-employed	5 (8)	
Other		4 (7)
**Condition profile *N* (%)**		
CHF	11 (18)	21 (38)
CHD	55 (92)	41 (73)
COPD	23 (38)	21 (38)
Diabetes	34 (57)	36 (64)
Two conditions	58 (97)	37 (66)
Three conditions	2 (3)	17 (30)
Four conditions	0	2 (4)

*N = 56 Belgium, three participants withdrew before completing their demographics and 1 participant was excluded from analysis due to not meeting the age criteria.

**Table 2.** Overview of themes and sub-themes.

Theme	Sub-theme (by participant group/s)
The work of triage	Holistic assessment (TNs)
	Education and motivation (TNs, PwMs)
	Time to care (TNs)
The benefits of triage support	Improved self-management (TNs, PwMs)
	Clinical interventions (TNs, PwMs)
	Reassurance (TNs, PwMs)
Tensions	Anxiety and burden (PwMs)
	Reconciling triage versus HCP opinion on alert thresholds (PwMs)
Relationship between PwMs and triage	(TNs, PwMs)

### The work of triage

#### Holistic assessment

Prior to talking to PwMs, whether for an alert or check-in call, triage nurses reported that they reviewed all ProACT data to give them the “full picture” of the person: *“You’re looking at the whole… You’d never look at any one thing in isolation”* (TN, IE); *“I suppose you’re looking at it and you go right down through, you know, all the little things, the walking, you know, their blood glucose”* (TN, IE). This allowed for a holistic assessment, which the nurses identified as important when working with people who have multiple conditions: *“Because there's so many comorbidities. You have to look at the whole”* (TN, IE). In addition to reviewing all data sources, the triage nurses also examined historical data from the previous week(s), to help them ask the right questions and reach care decisions. They reported looking for patterns and trends in the data. They correlated different types of data, such as symptom data across conditions, as well as symptom data with activity, sleep and self-reported wellbeing data: *“You knew, like, if their saturations* [blood oxygenation level for those with COPD] *were dropping and you could actually see that their steps are reduced over a few days as well”* (TN, IE). The nurses said the data allowed them to see the results of the strategies PwMs were putting in place to self-manage conditions and control symptoms: *“You’re able to marry up* [connect] *actually, as well the results of say the blood pressure, the glucose with whatever it is, so there's physical evidence to what they’ve done”* (TN, IE).

The nurses reported that holistically reviewing all data supported their decision-making when an alert occurred. The data helped in determining the course of action they advised the PwM to take, such as whether to adjust behaviour, contact the general practitioner (GP), or go to the Accident and Emergency Department: *“…we would know our clients, because of the information, and we can see that, okay, that is an anomaly, there we need to make a call”* (TN, BE). A factor identified as important in decision-making was not losing the person in the data. While the data was considered necessary in decision making, the nurses emphasised valuing the PwM's perception of how they were feeling, as well as the importance of assessing by listening; for example, someone with COPD might have a very low blood oxygen reading (which would create an alert) but would not be breathless while talking on the phone. The triage nurses would take both readings and the direct interaction with the PwM into account when responding to system alerts.

In addition to reviewing and following up on ProACT data or alerts and making regular check-in calls (including for those participants not alerting), triage nurses noted their workflow included educating and motivating participants and liaising with some family members and carers. Listening was frequently noted as an essential triage skill, with the ability to effectively make assessments and provide relevant information dependent on good listening.

#### Education and motivation

The triage nurses in Ireland spoke about educating PwMs, for example by helping them to understand their data. Such education included advising on strategies to manage high readings, tips on how to progress with activity goals, and how to access health and/or community services: *“You can have a little chat with them about their diet, you know, a glass of water and a 15-minute walk if you’re going to do that* [eat sugary foods] *will help with the blood glucose… Help to bring it back down”* (TN, IE). Triage nurses spoke about the importance of feeding information slowly to PwMs to allow them time to take it in, as well as constantly reinforcing messages. Triage nurses in Ireland also discussed how they provided support for PwMs with HCP visits, including support in preparing for the visit and discussion of the visit after it had happened. In terms of preparation, the triage nurses would advise PwMs to write questions for the visit and encourage them to ask their HCPs these questions: *“Ask about this and ask about that. Don't be afraid to ask questions. Get a pen and we’ll write them down”* (TN, IE). They also advised PwMs to bring their iPads to visits, to be able to show their ProACT data to their doctor. Following the visit, the PwMs often asked for help in understanding what HCPs had said: *“they’ve been to a hospital appointment with somebody or another healthcare professional and they’re coming back, and you might talk to them and they’d say, ‘Can you explain it to me?’”* (TN, IE).

Triage nurses in Belgium did not explicitly talk about educating participants, however PwMs in both countries spoke about how the triage nurses helped with understanding readings, thresholds for readings, medication intake and the effects of medication: *“I often ask them stuff, you know about diabetes or that. And about the readings and the blood sugars. And the same with the blood pressure. They explained top numbers and the bottom numbers and the relationship between the two of them and what to watch. It was all super”* (P053, M, 71, Diabetes + CHD, T4, IE); *“That I started to look after certain cases, yes. Supplementing oxygen to, as they say, put less pressure on my heart. I listened to that, to the messages I received* [from the nurses]*”* (P78, M, 77, COPD + CHD, T4, BE). A number of participants noted how this education from the triage nurses filled a gap: *“Well, those triage nurses I think explain more to me than my own GP”* (P047, M, 69, COPD + CHD, T4, IE).

As part of their calls, triage nurses also provided support in terms of motivating and encouraging PwMs to engage with self-management. The nurses reported doing this by nudging, reassuring, congratulating achievement and persevering: *“It's the congratulations without the patronising. Oh look, you’ve done really well there. I see you’ve been more active”* (TN, IE). In Ireland, triage nurses also noted the important role family and informal and formal (paid) carers play in persuasion and motivation of PwMs to self-manage. Triage nurses in Ireland also built up a relationship with some family members and formal carers over the phone, for example, if they called and the PwM wasn't available. Family members and carers often updated triage nurses on the PwM's status, while the nurses frequently asked family and carers to remind the PwM of certain things, such as to ask questions of HCPs during appointments. The nurses also noted that family were important as persuaders, for example, to help persuade a PwM to go to a hospital or the GP, if triage was recommending it.

#### Time to care

Triage nurses repeatedly mentioned how being involved in ProACT triage gave them time to care. Unlike a GP or hospital appointment, triage nurses didn't have an allocated length of time for their phone call with PwMs. This allowed them to listen to PwMs and build rapport: *“Sometimes all they want is somebody to listen to them. And we’ve time to do that”* (TN and IE). Triage nurses also noted they had time to review all ProACT data for PwMs, prior to their calls, which further supported their holistic approach to care. Similarly, the Belgian team expressed how their work on the ProACT triage helped them return to their nursing roots and how it gave them more time to react as it differed from their usual activities in which they provided emergency response via a home care alarm system, which included reacting to a flashing screen and typically directing an emergency response. During the trial, they could provide more holistic and patient-centred care, taking more initiative and time in their approach to the client.

### The benefits of triage support

PwMs and triage nurses across both countries noted various benefits of having the triage service alongside the digital self-management. Many PwM participants spoke of how the triage service was more than they expected it to be at the start of the trial. Participants expected they would be called and advised on what to do in case of a change in symptoms. However, the level of advice and education provided exceeded expectations and was seen as an extra benefit: *“It was more than I expected, it was better than I expected, they were very good ringing me up”* (P001, F, 76, COPD + CHD, T4, IE).

#### Clinical interventions

Triage nurses provided many examples of clinical interventions and medication adjustments that they facilitated during the trial. While this sometimes included the triage nurses calling an ambulance directly, it typically involved the provision of advice or recommendations for PwMs to visit their consultant or GP, for example to have a 24-h blood pressure monitor fitted. These interventions often resulted in medication adjustments: *“But there has been action, medical action, at the end of…* *You know, there has been an intervention, or their medication has been changed. They’ve been on* [24-h blood pressure] *monitors. They’ve been referred to specialist clinics. There's been so many, ‘Only for you, I wouldn't be talking to you because I went to my GP that day and they got an ambulance for me to the hospital’”* (TN, IE). This feeling was also shared by the Belgian triage team who said *“Some of them were satisfied* [with the call]*, because look it is good you gave me a call, cause most of them seem to think that they should wait. While we are of the principle that if for example on a Friday you have a heightened blood pressure, we rather have them call their GP* [for an appointment]*, even if it is only a call to discuss it with their GP, rather than wait for the next week Monday and to then have something more serious happen during the weekend”* (TN, BE). Nurses in both countries reported that participants were very willing to listen and would engage with the advice, for example, by asking questions on the call. It was noted as rare that PwMs refused to listen.

PwMs also spoke of experiencing positive clinical outcomes because of using ProACT and being monitored by the triage service. While in some cases, PwMs made the decision for themselves to visit their HCP because of their data, for others it was due to a recommendation from the triage nurses. During an interview with one PwM, their informal carer noted *“*[Triage nurse] *told me get her into a hospital straight away otherwise you’re going to have a body on your hands. Bring her out to the hospital and tell them the way she is. And I did that, and they kept her in…* *Yeah she* [nurse] *wanted to send an ambulance”* (spouse of P018, F, 73, Diabetes + CHD, T3. IE). Another common clinical outcome was new reports of changes in medication, noted from T3 onward. This occurred when a PwM was prompted to visit their HCP due to their data: *“Same day I told my GP and he changed my medication”* (P70, M, 71, CHD + CHF + COPD, T3, BE); “*They* [triage] *had suggested maybe to go and get my cholesterol checked and my cholesterol was a bit high. It had been going a bit high and then my medication was changed and it was as a result of ProACT”* (P039, F, 66, COPD + CHD, T4, IE).

#### Reassurance

PwMs overwhelmingly found the triage service to be positive, primarily due to the reassurance and support the triage nurses offered during the trial: *“Well, what I like the most about it is I feel that someone's watching out for you… And I know that they’ll ring me if they see anything wrong. So, in that, I think confidence in myself because I know I’m being watched”* (P039, F, 66, COPD + CHD, T2, IE). Most found it beneficial to know someone was looking out for them behind the scenes, alerting them if their readings were outside a normal range: *“The fact I knew someone was keeping an eye on it. And if that something was going out of kilter that I’d get a nudge. And that's all I’d need… The fact that there's someone with a better level of knowledge and who will contact me”* (P015, M, 82, Diabetes + CHD, T3, IE); *“I know now that someone looking at the data who is a professional, that they tell me what I see is a risk”* (P86, M, 70, Diabetes + CHD + CHF, T3, BE). Many also pointed out how they felt the triage nurses acted as an early warning system if anything were to go wrong. The triage not only had an impact on the PwM participants but also re-assured the live-in partners of the participants, as explained by P81: *“It has given my wife a feeling of reassurance. People are keeping an eye on me. For me… it is also a reassurance”* (P81, M, 68, CHD + CHF, T4, BE).

The fast response of triage in relation to alerts also contributed to participants’ feelings of reassurance. Many participants reported that the triage nurses would respond almost straight away following an abnormal reading: *“they were quick off the mark if the stats were low”* (P030, F, 77, COPD + CHD, T4, IE); *“that triage nurse, automatically if she sees any glitch in it or any fault, she's on to me straight away”* (P047, M, 69, COPD + CHD, T4, IE).

Triage nurses felt the service gave confidence to PwMs, that they saw it as a ‘safety net’. The triage nurses also always followed up with PwMs, for example after an alert, medical intervention by their usual HCP, or if they were feeling ill. Staff felt this was very much appreciated by PwMs, who often reported little follow-up from their HCPs. The education and encouragement provided by the nurses to PwMs were also seen as giving PwMs confidence to self-manage. While some PwM participants, particularly by the end of the trial, felt adept enough at self-managing to know themselves when they should do something about a reading, the majority still welcomed the triage support, in case they were to miss something: *“It gives you a kind of a safer feeling that if there is something wrong that you missed… there are people on the other end of the computer who won't miss it and they will ring you”* (P042, 70, M, Diabetes + CHD, T4, IE); *“Yes, that is a feeling of safety of course. That when you are home alone, then it is good to know..”* (P66, F, 69, Diabetes + CHD, T4, BE).

#### Improved self-management

It is unclear whether improvements were a result of PwMs using the ProACT platform and actively monitoring and reviewing data, or the education, advice and feedback provided by the triage nurses or a combination of both the technology and triage support. Regardless, triage nurses observed that participants were alerting less over time, indicating that PwMs were becoming more adept at self-managing their conditions and symptoms: *“Those that were alerting at the beginning are not alerting now…* *They had alerts going off left, right and centre at the beginning and they don't have alerts going off now”* (TN, IE); *“Now they’re managing their conditions so much better”* (TN, IE). The nurses reported that this resulted in fewer hospital interventions at later stages of the trial: *“I think early on, we probably had a lot more intervention from the point of view of getting them into a hospital”* (TN, IE); as well as improvements in overall quality of life due to less ill time: *“Like you can see in most of them, you can actually see an improvement in their quality of life”*; *“he has transformed his whole spirit, his wellbeing and everything has been great”* (TN, IE). Alongside this, participants were informing triage that they were having less healthcare utilisation in terms of hospital stays and triage nurses attributed this to ProACT helping PwMs to stay at home, by acting as an early catch for problems: *“You’re catching them* [through ProACT]*, so if they go to their GP for an antibiotic, they’re not ending up in hospital and that's huge because it's a win-win for everybody”* (TN, IE).

It was felt by the triage teams that, over time, PwMs demonstrated insight into the management of their conditions, for example, PwMs would tell triage staff what they had done or eaten to trigger the alert. PwMs could also pinpoint what had caused a particular alert that had subsequently triggered a triage call: *“One of the girls* [nurses] *rang one day on a Monday. I knew it was coming. My sugars were at 22 over the weekend and she rang me* (laughs)*, she wanted to know why it was so high and I knew straight away what it was – I binged at the weekend”* (P042, 70, M, Diabetes + CHD, T4, IE). PwMs also told the nurses that they were ‘testing’ how their behaviours impacted on their symptoms, which triage felt helped with providing insight (e.g., how eating something with high sugar content impacted blood glucose readings).

### Tensions

Despite the various benefits of the triage service that were reported, some tensions between PwMs and the triage service were apparent from interviews with PwMs, including increased anxiety due to being monitored by triage, and differences of opinion between triage nurses and PwMs’ HCPs.

#### Anxiety and burden

While the feedback on the triage service was mostly positive, a small number of participants reported that the service could cause them anxiety or panic. P032 found anticipating a call from triage as a result of a high blood pressure reading stressful: *“And I’ve a phone call* [from triage] *and they’re very nice… But it stresses me… That is the worst part, I have to say, and I know they’re doing their job, but it's just you have this idea Big Brother's watching”* (P032, F, 76, Diabetes + CHD, T2, IE). This participant opted to use a personal blood pressure cuff that was not connected to the ProACT system so that if she had a high reading, there would be no alert triggered with triage. P036, in particular, felt quite anxious about getting a call from triage, as he knew this meant that his symptoms were out of range: *“I found it really frightening. It actually brought on the symptoms”* (P036, 72, M, COPD + CHD, T4, IE). Despite reporting being ‘scared’ of triage calls, he could see the benefit of the service: *“With all due respect to the triage nurse.. despite the scares, it was nice to know that there was somebody out there that cared”*. P036 found it difficult to balance the anxiety created by a potential triage call with the benefit of knowing someone was looking out for him and this ultimately led him to use ProACT less often as the trial progressed. A small number of participants also reported additional negative opinions of triage, including nurses calling at inconvenient times, being over-cautious and calling too often: *“With the blood pressure if it was a bit too low or too high, I would receive a phone call… But that then has… they called too many times. […] I then said: “There is nothing wrong, you don't have to call every day”* (P77, 66, COPD + CHD, T4, BE); *“Look, the problem is that I do not always have my mobile phone next to me. I have noticed that you tried to call me 3 or 4 times but I was gone. I am not someone who constantly walks around with a telephone”* (P61, M, 70, COPD + Diabetes + CHD, T3, BE).

#### Reconciling triage versus HCP opinion on alert thresholds

Several participants spoke about how they discussed their readings with HCPs during clinical visits. Triage nurses often encouraged PwMs to bring their iPad with their data to such visits. Some participants reported HCPs being less concerned about their readings than triage: *“But on two occasions now, when they* [triage] *did ring me about my readings, when I go to the hospital they didn't seem to be that concerned in a way.. The ProACT* [triage] *told me to bring in my iPad and show them for the past week the way it was. And the triage felt that they weren't happy with it. It was very low. But in the hospital, they felt that where it was and me in the condition that I have with COPD and on dialysis, that it was nearly normal”* (P026, M, 75, COPD + CHD, T2, IE); *“*[they called] *about my pulse. That it was too low. But that does not matter that it is too low. The cardiologist knows and he is not worried”* (P63, M, 76, Diabetes + CHD, T4, BE). For others, this prompted a discussion on personalising alert thresholds for the individual: *“The blood pressure, I spoke with my doctor about it and he said to me ‘look you’re in your early 80*′*s’, he said ‘you should look at 150 over 100’, not 140 over 80 that I was doing* [the default alert threshold set in the ProACT system for high blood pressure]*”* (P015, M, 82, Diabetes + CHD, T2, IE). Personalisation of alerts also led to less over-alerting, which was appreciated by many PwMs *“Triage will not call you again when it is 14. They know that 14 for you is normal. So I think that is good that they adjusted that for me”* (P102, M, 69, CHD + CHF, T3, BE).

### The relationship between PwMs and triage

A large theme to emerge concerned the positive relationship that developed between many PwMs and the triage nurses over the course of the trial. For some PwM participants, the triage service appeared to function as an emotional and social support. PwMs felt the triage nurses truly cared about their wellbeing. Participants felt they got to know nurses personally, and that the nurses came to know them and their health readings. The majority of participants in Ireland commented on how the nurses took the time to chat with them, enquire about their health and, overall, found the phone calls to be a pleasant experience. Participants perceived that the triage service also gave social support and provided social connection: “*They just ring up and they’re chatty*” (P057, M, 83, CHF + CHD, T2, IE); “*I mean you can vent and say anything you want* [to triage] *and that's what I love about it*” (P039, F, 66, COPD + CHD, T2, IE). PwMs felt that triage listened, were perceptive and always followed up: *“Even an off-the-cuff remark, they didn't miss it and they were perceptive and would mention it, bring it up again or bring it up casually or just reinforce it nicely”* (P041, M, 69, Diabetes + CHD, T4, IE). The participants in Belgium were positive about their experience of the triage, however, they did not express their relationship with the triage nurses in the same effusive language. The focus in Belgium was more on the effectiveness: *“The triage worked well, yes I have couple of times received a red signal, and I have received a couple of calls,* [to discuss that] *this and that is not okay, so that is very positive I think”* (P85, M, 81, Diabetes + CHD, T4, BE).

Triage nurses in both countries also spoke of the relationships that had formed between them and the PwMs. Developing a rapport with PwMs was considered critical to keep the person calm, help them relax, and to build up trust with the PwM. Such trust in turn helped the nurses gather the information they needed to make decisions and motivate PwMs, showing that the nurse cared. The triage nurses spoke of how they achieved rapport by ensuring their tone is informal and calm, rather than antagonistic, and by ‘not acting like a school teacher’. In Ireland, nurses reported logging personal anecdotes about PwMs in the SIMS-triage system that they could then return to on the next call: *“We can restart the conversation where it left off and they just feel like we know them”* (TN, IE). This also helped PwMs to perceive continuity in their care*.* As a result, the triage staff believed the majority of PwMs enjoyed their ‘chats’ with the nurses and it helped PwMs to speak openly and honestly (e.g., about the cause of alerts). In Belgium, the nurses commented that the PwMs developed a special rapport with specific nurses and would sometimes ask to talk to a specific person: *“but they would still ask for that specific person with whom they had the most contact”* (BE).

Closely linked to rapport is the importance of *trust* which was noted as important to encourage participants to speak openly, but primarily so that the PwM knows that the nurse will provide the best advice for them. One nurse noted how many older people fear going to hospital and, therefore, were concerned about being referred to hospital by triage: *“Because a lot of the older people, they’re terrified you’re going to send them to hospital. So they’re actually going to lie to you on the phone”* (TN, IE)*.* Despite this, it was evident from the PwM interviews that there was an awareness that early interventions could help to avoid hospitalisation.

The admiration the Irish nursing staff felt for the PwMs was evident throughout, with statements such as *“They’re incredible really”; “They’re brilliant”.* The nurses expressed admiration for the PwMs’ engagement with ProACT and their positive outlook on life despite managing multiple conditions: *“It seems to be that there's a huge world out there and regardless of what you have, whatever you’re carrying on your back, COPD, diabetes, you can have a full life”* (TN, IE). Admiration for PwMs was not identified as a theme in the triage focus groups and interviews in Belgium.

## Discussion

Digital health technologies hold great potential to support older adults living with multiple chronic conditions to self-manage at home, enabling more patient-centred care. There is a large body of research on how such technologies can support and empower patients to self-manage single chronic diseases independently.^11,12^ However, given the complexity of multimorbidity management, coupled with changes that occur as part of the normal ageing process, human support alongside digital self-management may be necessary to sustain successful self-management. The importance of clinical or expert support for those managing chronic conditions has been noted and can result in greater self-efficacy and adherence to self-management.^10,18,19^ However, HCPs typically only see patients at scheduled appointments – or when a condition exacerbation requires urgent medical attention. HCPs have also expressed a lack of time for reviewing patient data from remote monitoring systems^12,26^ and a desire for patients to understand how to respond to exacerbation alerts from digital health technologies themselves,^7^ which in itself requires education and training that typically is not available to patients. If older adults with multimorbidity are to effectively self-manage, new models of patient-centred care are required to support each of these challenges.

This article presents findings from older adults with multimorbidity and triage nurses across Ireland and Belgium, who used the digital health platform ProACT for approximately one year to support the management of multiple conditions. Our findings indicate that triage nurses played a significant supporting role, not only responding to alerts but educating and motivating participants, acting as social support, as well as providing a holistic approach to care through assessing the person as a whole rather than considering individual chronic conditions in isolation of others. For the most part, the same themes were identified in Ireland and Belgium. One notable difference is the type of relationship between the participants and the triage nurses in each country. In Ireland, the relationship appeared to be more personal than in Belgium, with both participants and triage nurses using more effusive language to describe this relationship. The provision of education to support PwMs self-managing was also a stronger sub-theme in Ireland than in Belgium.

In the remainder of the discussion, we frame our main findings within the context of Hudon et al.'s. proposed framework for patient-centred care,^1^ specifically the dimensions of biopsychosocial perspective, therapeutic alliance and sharing power and responsibility.

### Biopsychosocial perspective

Our study findings indicate that through a combination of reviewing data across multiple conditions, in the ProACT platform, and taking the time on calls to actively listen and assess, the triage nurses considered the participant as a whole and any advice provided to participants was based on this full picture. PwMs typically do not report experiencing a sufficient level of integrated care. They have to consult with various HCPs for their different conditions, which often means patients receive conflicting information in relation to what they should prioritise and what self-management strategies they should implement.^10,35^ Education and advice provided by the triage nurses in our study considered the whole person and their current health and wellbeing status, allowing for a more personalised and integrated response.

Both triage nurses and PwMs mentioned how participants would bring their iPad with the ProACT CareApp and their data to clinical visits, allowing them to share the full picture of their health and wellbeing with doctors. Patients with single diseases have complained about having to compile data from disparate data streams across different self-management applications so that they can share their data in an organised way with healthcare providers.^7^ Patients in that study called for a way to combine symptom data with goals and how they are feeling physically, emotionally and mentally (ibid). Therefore, having one platform, such as ProACT, where all relevant data is available and accessible to different stakeholders can further support patients in experiencing more holistic and integrated care.

Finally, a small number of participants reported feeling anxious due to ‘surveillance’ by the triage team. Digital interventions where clinical monitoring is an inherent part of the solution design, may need to consider this. Whether anxiety was related to a fear of calls potentially leading to hospitalisation, knowing that readings were being monitored, or more specifically receiving reminders of such monitoring when alerts resulted in a call from the triage nurses, requires additional examination.

### Therapeutic alliance

The quality of the relationship between patients and HCPs is a significant determinant of positive health outcomes.^5^ However, therapeutic alliance or the patient-doctor relationship is often not experienced as therapeutic for those with multimorbidity due to various issues and barriers including poor access, poor communication and lack of trust^10,12,21–23^ A key theme to emerge in our findings related to the positive relationships between triage nurses and the participants of the trial. Triage nurses provided not only clinical support but also social support, a strong enabler of self-management,^10^ where social support includes emotional support (e.g., empathy), appraisal (e.g., information for self-evaluation) and information (e.g., advice, guidance).^36^ The triage nurses put effort into establishing a relationship, highlighting the importance they placed on rapport in building trust. Such efforts from HCPs can help provide comfort and establish trust, which in turn plays an important role in patients determining if and how they want to be involved in their care and motivating patients in their self-management.^7^ Studies on trust between HCPs and patients with multiple chronic conditions are lacking, however.^21^

A likely factor in building rapport and trust between triage nurses and participants of the trial was the frequency and nature of the contact that participants perceived they had with the triage service because the triage nurses had ‘time to care’. Participants reported rapid responses to alerts, having ‘chats’ with the nurses, feeling that their conversations and care were followed up, and receiving advice and education to support their digital self-management. This is in contrast to the barriers PwMs often report with respect to their relationships with their usual HCPs, as outlined in the introduction to this article. Continuity of care is a key factor in the satisfaction of older patients with their HCPs and has been identified as a missing ingredient in the primary care management of multimorbidity.^37^ Higher continuity of care provision for community-dwelling older adults is also associated with reduced preventable hospitalisations, particularly for patients with CHF and COPD.^38^

### Sharing power and responsibility

Shared decision-making is a core aspect of patient-centred care, whereby patients and their HCPs collaborate, discuss and agree on a care plan and where patients have the necessary expertise to take responsibility for their care.^39^ However, true collaboration requires a significant shift from the current types of relationships that exist between HCPs and their patients, where patients report poor access to HCPs,^10^ who in turn report feeling burdened by having to monitor patient data.^26^ It has been noted that unless health systems and providers are willing and able to change, the self-management efforts of patients may have limited success.^10^

To ensure patients have sufficient knowledge to enable more sharing of power and responsibility, and to self-manage effectively, education and training are necessary. However, lack of education is often cited as a barrier to effective self-management of multimorbidity.^10,12^ During the ProACT trial, participants were responsible for their own self-management and were supported in this through training sessions with the technology and through provision of education on condition management, general wellbeing and technology usage through the CareApp. The triage nurses reinforced the education provided through ProACT (e.g., explaining the ‘top and bottom numbers’ of a blood pressure reading) and helped put it into context. Furthermore, they supported the PwM in having a more equal relationship with their HCPs by helping them prepare for clinical visits and encouraging them to share their data with their HCPs. Responses to alerts were also negotiated between triage nurses and PwMs, where discussions with PwMs during alert follow-up calls, allowed for the context of an alert, as explained by the PwM, to be brought to bear on the interpretation of the alert. Furthermore, these discussions sometimes resulted in the establishment of revised alert parameters as negotiated between the PwM and the triage team.

Due to a lack of integrated care for those with multimorbidity, the person is often required to be the coordinator of their own care, which is challenging. The model tested through the ProACT trial was the PwM (either with or without a carer) being responsible for care coordination, but with support from the triage nurses - in understanding exacerbations and alerts, encouragement to bring their data to clinical visits to discuss with HCPs, and advice on preparing for clinical visits. This resulted in the person having more confidence during HCP visits to ask questions and voice their opinion on what they wanted. This also provided peace of mind as they knew they would get a call if there was an alert and they would have someone to talk this through with, without feeling they were taking up someone's time.

Some research cautions against the provision of too much support for those self-managing, as it can result in patients taking less responsibility for self-management. For example, Morton et al.^26^ have noted that patients tend to rely more on HCPs if they receive regular feedback from the HCP. The problem can be exacerbated when HCPs respond to alerts, as patients then rely on HCPs to identify and respond to alerts rather than acting on them themselves. Our findings somewhat contradict this. Participants in our study spoke of the triage nurses as ‘backup’ and providing reassurance, rather than relying on them to identify issues. Indeed, by the end of the trial, PwMs reported feeling confident in self-management, understanding their data, but noted that they still welcomed the triage support. A potential reason for this difference is that patients in Morton et al.'s study were managing a single chronic condition. However, the complexity of multiple chronic conditions may justify clinical oversight. Furthermore, personal condition support needs vary as disease trajectories change over time and as people continue to age.

As noted by Yardley et al.^19^ and others, using digital self-management systems without human support can negatively impact engagement, resulting in drop-out and non-usage attrition. Findings on engagement with self-management during the ProACT trial have been reported elsewhere^31^ and demonstrate that on average, participants took between two and three health readings daily. A total of eight participants withdrew from the study early in Ireland, while 11 withdrew in Belgium, primarily due to ill health. Yardley et al.^19^ suggested that research should examine at what point or points of a self-management journey human support adds value to a digital health intervention. However, it could be argued given the complexity of multimorbidity, the potential for serious exacerbations for various conditions, and the dynamic nature of diseases combined with the ageing process, that sustained human support for digital self-management has value.

### Practical implications

The importance of patient-centred care from a biopsychosocial perspective, as well as sharing power and responsibility as a therapeutic alliance are accepted as crucial to the successful management of chronic conditions, whether from the perspective of the patient, the HCP or the wider social system. However, in practice, many healthcare systems and healthcare providers are not in a position to provide a level of patient-centred care that patients desire or need for effective self-management, or indeed that is recommended in the literature, thus leaving a gap in integrated and continuous care provision. Research has suggested the role of a care coordinator, such as the triage nurses in this study, can be pivotal in condition self-management.^10,19^ Our findings indicate that remote clinical triage nurse monitoring, providing care through a combination of a digital health platform and telephone support, can fill this gap. How such an approach can be implemented into health systems would require a shift towards a community/person-centred driven model of digital integrated care, ensuring that individuals have access to personalised and tailored support for their health and wellbeing management at home.

### Limitations

The larger research trial from which these findings were identified, was not specifically designed to examine the impact of triage nurse monitoring on self-management of multimorbidity by older adults. The triage nurse element evolved over the course of the trial from exclusively responding to reading alerts at the start of the trial, to making monthly check-in calls to participants as the trial progressed. A deeper examination of the topic may have been possible if such exploration had been part of the initial research design. Nonetheless, the rich longitudinal and multi-stakeholder data collected, as well as the close engagement by researchers with participants, facilitated recognition of the value of examining the topic in the data collected. Likewise, despite the limitations in representative validity of findings, from the small number of triage nurses (seven in total across both trial sites), two different clinical organisations are represented along with 120 older PwMs from two countries who have provided valuable insight, indicating a direction for additional research to further explore the findings presented in this paper.

## Conclusion

Digital health technologies hold great potential to support the self-management of multiple chronic conditions and empower people to be at the centre of their care. However, to achieve patient-centred care, it is important that healthcare providers consider the whole person, support shared decision-making and responsibility, and that patients perceive a positive relationship with their providers. This study aimed to identify the role that a nurse-led telephone triage service, supported by the use of home-based digital technology, can play in supporting older people with multiple chronic conditions to digitally self-manage at home. This work took place within the context of the larger ProACT proof-of-concept trial, whereby older adults with multimorbidity used the digital health platform ProACT to self-manage their conditions over a period of approximately one year, with support from clinical triage nurses. Thematic analysis of interviews and focus groups with both PwM and triage nurse participants revealed four main themes; the work of triage nurses in supporting PwMs, the benefits of triage support, tensions and the relationship between PwMs and triage nurses. Findings were discussed within the context of Hudon et al.'s patient-centred care framework^1^ and indicate that patient-centred care was achieved, with both PwM and triage participants reporting positive experiences, relationships and several benefits of the triage support alongside digital self-management. Future work should further explore the potential benefits of healthcare systems having a role dedicated to clinical support for digital self-management.

## References

[1] Hudon C Fortin M Haggerty JL, et al. Measuring Patients’ perceptions of patient-centered care: a systematic review of tools for family medicine. Ann Family Med [Internet] 2011; 9: 155. [cited 2021 Aug 31] Available from: /pmc/articles/PMC3056864/. 10.1370/afm.1226 PMC3056864 21403143

[2] Boyd CM Fortin M. Future of multimorbidity research: how should understanding of multimorbidity inform health system design? Public Health Rev 2010; 32: 451–474.

[3] van der Heide I Melchiorre MG Quattrini S, et al. Innovating care for people with multiple chronic conditions in Europe: an overview [Internet]. 2015 [cited 2020 Jul 30]. Available from: www.nivel.eu

[4] Multimorbidity: a priority for global health research. Academy of Medical Sciences [Internet]. 2018 [cited 2020 Jul 30]; (April). Available from: https://acmedsci.ac.uk/file-download/82222577

[5] Stewart M Brown JB Weston WW, et al. Patient-centered medicine: transforming the clinical method. Sage Publications, 1995. 267.

[6] Mead N Bower P. Patient-centredness: a conceptual framework and review of the empirical literature. Soc Sci Med 2000; 51: 1087–1110. 11005395 10.1016/s0277-9536(00)00098-8

[7] Pichon A Horan E Massey B, et al. Divided we stand: the collaborative work of patients and providers in an enigmatic chronic disease ACM reference format. In: Proceedings of the ACM on Human-Computer Interaction [Internet]. 2020 [cited 2021 Aug 23];4(CSCW3). Available from: 10.1145/3434170 PMC8112593 33981961

[8] Barlow J Wright C Sheasby J, et al. Self-management approaches for people with chronic conditions: a review. Patient Educ Couns 2002; 48: 177–187. 12401421 10.1016/s0738-3991(02)00032-0

[9] Audulv Å Asplund K Norbergh KG. The integration of chronic illness self-management. Qual Health Res [Internet] 2011; 22: 332–345. [cited 2021 Sep 24]; Available from: https://journals.sagepub.com/doi/10.1177/1049732311430497, http://dx.doi.org/101177/1049732311430497 10.1177/1049732311430497 22167155

[10] Liddy C Blazkho V Mill K. Challenges of self-management when living with multiple chronic conditions: systematic review of the qualitative literature. Can. Fam Phys 2014; 60: 1123–1133. PMC4264810 25642490

[11] Haggerty JL. Ordering the chaos for patients with multimorbidity. BMJ [Internet] 2012; 345: e5915. [cited 2021 Aug 19]; Available from: https://www.bmj.com/content/345/bmj.e5915 10.1136/bmj.e5915 22960377

[12] Doyle J Murphy E Kuiper J, et al. Managing multimorbidity: identifying design requirements for a digital self-management tool to support older adults with multiple chronic conditions. In: Conference on Human–Factors Computer Systems - Proceeding 2019; 1–14.

[13] World Health Organisation. Adherence to long-term therapies: evidence for action. 2003 [cited 2021 Aug 19]; Available from: http://apps.who.int/iris/bitstream/handle/10665/42682/9241545992.pdf?sequence=1

[14] Fernandez-Lazaro CI García-González JM Adams DP, et al. Adherence to treatment and related factors among patients with chronic conditions in primary care: a cross-sectional study. BMC Fam Pract [Internet] 2019; 20. 10.1186/s12875-019-1019-3 PMC6744672 31521114

[15] Burgess ER Reddy MC Davenport A, et al. “Tricky to get your head around”: Information work of people managing chronic kidney disease in the UK. In: Conference on human factors in computing systems - proceedings [Internet]. ACM; 2019 [cited 2019 May 17]. Available from: 10.1145/3290605.3300895

[16] Blondon K Klasnja P Coleman K, et al. An exploration of attitudes toward the use of patient incentives to support diabetes self-management. Psychol Health [Internet] 2014; 29: 552–563. Available from: http://www.ncbi.nlm.nih.gov/pubmed/24256342 10.1080/08870446.2013.867346 24256342

[17] Visser L Shahid S Al Mahmud A. Point-of-care testing for diabetes patients: investigating diabetes management by older adults. In: Conference on human factors in computing systems - proceedings. Association for computing machinery; 2014. p. 1645–1650.

[18] Nunes F Fitzpatrick G Kyng M, et al. Self-care technologies in HCI: trends, tensions, and opportunities. In: ACM Transactions on Computer-Human Interaction [Internet] 2015; 22. [cited 2018 Sep 6]. Available from: 10.1145/2803173

[19] Yardley L Spring BJ Riper H, et al. Understanding and promoting effective engagement with digital behavior change interventions. Am J Preventive Med [Internet] 2016; 51: 833–842. 10.1016/j.amepre.2016.06.015 27745683

[20] Chewning B Bylund C Shah B, et al. Patient preferences for shared decisions: a systematic review HHS public access. Patient Educ Couns 2012; 86: 9–18. 21474265 10.1016/j.pec.2011.02.004 PMC4530615

[21] Ongwere T Clawson J Cantor GS, et al. Design and care for discordant chronic comorbidities: a comparison of healthcare providers’ perspectives. In: Proceedings of the EAI international conference on pervasive computing technologies for healthcare [Internet]. 2020; (October).

[22] Ongwere T Cantor G Martin SR, et al. Design hotspots for care of discordant chronic comorbidities: patients’ perspectives. In: Proceedings of the 10th Nordic Conference on Human Computer Interaction (NordiCHI). 2018. p. 571–583.

[23] Berry ABL Lim CY Hirsch T, et al. Supporting communication about values between people with multiple chronic conditions and their providers. In: Conference on human factors in computing systems - proceedings [Internet]*.* ACM; 2019 [cited 2019 May 15]. Available from: 10.1145/3290605.3300700

[24] Lim C Berry ABL Hirsch T, et al. It just seems outside my health”: How patients with chronic conditions perceive communication boundaries with providers. Des Interact Syst 2016; 1172. 10.1145/2901790.2901866 PMC5553690 28804790

[25] Colorafi K Evans B Lamb G. Engagement with the plan of care among older adults with multiple cardiac diagnoses. Qual Health Res 2021; 31: 1234–1246. 33769160 10.1177/10497323211001344

[26] Morton K Dennison L May C, et al. Using digital interventions for self-management of chronic physical health conditions: a meta-ethnography review of published studies Europe PMC funders group. Patient Educ Couns 2017; 100: 616–635. 28029572 10.1016/j.pec.2016.10.019 PMC5380218

[27] Fairbrother P Ure J Hanley J, et al. Telemonitoring for chronic heart failure: the views of patients and healthcare professionals – a qualitative study. J Clin Nurs [Internet] 2014; 23: 132–144. [cited 2021 Aug 19] Available from: https://onlinelibrary.wiley.com/doi/full/10.1111/jocn.12137 10.1111/jocn.12137 23451899

[28] Urowitz S Wiljer D Dupak K, et al. Improving diabetes management with a patient portal: qualitative study of a diabetes self-management portal. J Med Internet Res [Internet] 2012; 14, [cited 2021 Aug 19]. Available from: /pmc/articles/PMC3510725/10.2196/jmir.2265 PMC3510725 23195925

[29] Vassilev I Rowsell A Pope C, et al. Assessing the implementability of telehealth interventions for self-management support: a realist review. Implementation Sci [Internet] 2015; 10. [cited 2021 Aug 19] Available from: http://www.bristol.ac.uk/healthlines/. 10.1186/s13012-015-0238-9 PMC4424965 25906822

[30] Lenferink A. Self-management exacerbation action plans in patients with chronic obstructive pulmonary disease THE COPE-III STUDY. 2017. 260 p.

[31] Doyle J Murphy E Gavin S, et al. A digital platform to support self-management of multiple chronic conditions (ProACT): findings in relation to engagement during a one-year proof-of-concept trial. J Med Internet Res [Internet] 2021; 23: e22672. Available from: https://www.jmir.org/2021/12/e22672. 10.2196/22672 PMC8717138 34914612

[32] Dinsmore J Hannigan C Smith S, et al. A digital health platform for integrated and proactive patient-centered multimorbidity self-management and care (ProACT): protocol for an action research proof-of-concept trial. JMIR Res Protoc [Internet] 2021; 10: e22125. https://www.researchprotocols.org/2021/12/e22125. Available from. 10.2196/22125 PMC8717136 34914613

[33] Braun V Clarke V. Using thematic analysis in psychology. Qual Res Psychol 2006; 3: 77–101.

[34] Lincoln YS Guba EG. Naturalistic Inquiry SAGE Publications Ltd [Internet]. 1985 [cited 2022 Jan 11]. Available from: https://uk.sagepub.com/en-gb/eur/naturalistic-inquiry/book842

[35] Caldeira C Gui X Reynolds TL, et al. Managing healthcare conflicts when living with multiple chronic conditions. Int J Hum Comput Stud [Internet] 2020; 145: 102494. [cited 2020 Jul 31] Available from: 10.1016/j.ijhcs.2020.102494

[36] Cohen S. Social relationships and health. Am Psychol [Internet] 2004; 59: 676–684. [cited 2021 Sep 8] Available from: /record/2004–20395-002. 10.1037/0003-066X.59.8.676 15554821

[37] Engamba SA Steel N Howe A, et al. Tackling multimorbidity in primary care: is relational continuity the missing ingredient? Br J General Pract Royal College of General Practitioners 2019; 69: 92–93. 10.3399/bjgp19X701201 PMC6355272 30705019

[38] Nyweide DJ Anthony DL Bynum JPW, et al. Continuity of care and the risk of preventable hospitalization in older adults. JAMA Internal Med [Internet] 2013; 173: 1879–1885. [cited 2022 Jun 29] Available from: https://jamanetwork.com/journals/jamainternalmedicine/fullarticle/1738715 10.1001/jamainternmed.2013.10059 PMC3877937 24043127

[39] Barry MJ Levitan SE. Billingham -Valerie. Shared Decision Making-The Pinnacle of Patient-Centered Care Nothing about me without me. 2012.

